# Conductivity of Insulating Diblock Copolymer System Filled with Conductive Particles Having Different Affinities for Dissimilar Copolymer Blocks

**DOI:** 10.3390/polym12081659

**Published:** 2020-07-25

**Authors:** A.I. Chervanyov

**Affiliations:** Institut für Theoretische Physik, Westfälische Wilhelms-Universität Münster, Wilhelm-Klemm-Strasse 9, 48149 Münster, Germany; chervany@uni-muenster.de

**Keywords:** diblock copolymers, fillers, conduction, 83.80.Uv, 82.35.Np

## Abstract

We investigate the electrical response of the insulating diblock copolymer system (DBC) filled with conductive spherical fillers depending on the affinities of these fillers for copolymer blocks and the interaction between fillers. We demonstrate that the contrast (difference) between the affinities of the fillers for dissimilar copolymer blocks is a decisive factor that determines the distribution of these fillers in the DBC system. The distribution of filler particles, in turn, is found to be directly related to the electrical response of the DBC-particle composite. In particular, increasing the affinity contrast above a certain threshold value results in the insulator-conductor transition. This transition is found to be caused by the preferential localization of the fillers in the microphases of the DBC system having larger affinity for these fillers. The effect of the interaction between fillers is found to be secondary to the described effect of the affinity contrast that dominates in determining the distribution of fillers in the composite. This effect of the inter-particle interactions is shown to be significant only when the affinity contrast and filler volume fraction are sufficiently large.

## 1. Introduction

Composites comprised of polymeric insulating materials filled with conductive fillers have broad spectrum of applications ranging from sensors and actuators [1,2,3,4,5,6,7] to micro-electromechanical systems [8,9] and electronic packaging [10,11,12]. The main advantage of a soft polymer matrix as a host system for fillers relative to its hard counterpart is that this matrix can be easily adjusted to required conditions by changing its shape and volume. In particular, host soft polymer matrix has high responsiveness to external stimuli such as shear and stress, which opens a route towards sensing applications [13,14].

Despite its documented practical importance [15], electrical conductivity of composites comprised of *inhomogeneous* polymer host systems and conductive nano-particles has not yet received due theoretical attention. Few theoretical studies [13,16,17] address homogeneous insulating host polymer systems containing conducting fillers. In these models, the role of the host polymer matrix is restricted to insulating the contacts between the conductive fillers. This insulating effect is used to induce the conductor-insulator transition that occurs when the fraction of conductive fillers in a composite reaches a certain percolation threshold. This threshold can be shifted [2,3,13,14] by applying stress to a polymer-particle composite that causes restructuring the conductive filler network formed in this composite. This restructuring results, in turn, in switching among conductivity mechanisms (e.g., constriction and hoping) and changing overall conductivity of the composites in a wide range [13]. The relation between the mechanical deformations and the conductivity of composites can be used [13], in particular, in piezoresistive soft mechanical-electrical sensors.

The key mechanism that affects the electrical properties of the described polymer-particle composites is the assembly of particles that form conductive clusters inside this composite [18]. The above described previous work relies on affecting the electrical response of composites by indirectly modifying the distribution of fillers in this composite. A desirable density structure of fillers inside a composite is produced by changing physical structure of this composite through applying external stimuli such as permanent of periodic mechanical load (stress) [13,14] or shear [19]. In the present work we explore a qualitatively different route to governing the conductivity of polymer-particle composites by changing the compositional structure of a host polymer system. A host polymer matrix most suitable for this purpose is the diblock copolymer system (DBC) that is known to micro-phase separate and assume different morphologies in response to changing temperature or pressure [20,21]. Depending on the copolymer composition, the thus formed micro-phases can have well-defined geometrical form of lamellae, spherical or cylindrical domains having a typical size of several hundreds of nanometers. The microphase separation of DBC, in turn, can cause [22,23] the redistribution of fillers through the formation of well-defined domains enriched with fillers. We demonstrate that DBC system containing the described filler-rich domains have larger conductance relative to the homogeneous counterpart of this system.

By combining the Monte Carlo simulations and phase field approach we demonstrate that the distribution of particles essentially depends on the contrast between affinities of these particles for dissimilar copolymer blocks. When the above affinity contrast is sufficiently large, the filler particles are found to be preferentially localized within polymer domains that have larger affinity for these particles. Note that this observation is in agreement with our previous results independently obtained by the self-consistent field theory [23]. The described enthalpic effect causes an essentially non-uniform distribution of particles in the DBC system resulting in the formation of the aforementioned filler-rich domains. The local density and probability of contacts among the filler particles placed within the selective DBC domains is increased, which promotes an increase in the total conductance of the composite. We therefore prove that the conductance of the DBC-particle composites can be varied in a wide range by changing the morphology of this composite and affinities of the fillers for copolymer blocks.

An additional effect on the distribution of fillers relies on the tendency to screen energetically unfavorable contacts between dissimilar copolymer blocks. As a result, the fillers tend to be localized within the interfaces between different polymer phases where such unfavorable contacts prevail. In contrast to the above described enthalpic effect that stems from different adsorption strengths of filler particles for copolymer blocks, this effect is of osmotic (excluded volume) origin. In the incompressible DBC system having relatively large degree of segregation between different polymer blocks considered in the present work, the osmotic screening effect is found to be less pronounced relative to the enthalpic effect. The relative significance of the described osmotic and enthalpic effects can vary for different relations between strengths of the interaction between the dissimilar copolymer blocks and that between copolymer blocks and particles. We surmise that when the particles have close affinities for the copolymer blocks, their distribution is strongly affected by the osmotic effect that must be therefore always taken into account.

It is also worth noting that most of the previous experimental and theoretical work deals with non-spherical fillers having large aspect ratio (e.g., carbon nanotubes). This increased interest to elongated fillers is due to the fact that assemblies of these fillers [18] have a much lower electrical percolation threshold relative to those of spherical fillers. Many practical applications, in contrast, rely on employing spherical fillers. Common examples are provided by carbon black fillers used in tire technology [24] and spherical silver fillers used in microelectronic applications [16]. In particular, spherical fillers can be better compatibilized with a polymer matrix and they form clusters in heterogeneous polymer systems more easily. To suite engineering applications, it is therefore imperative to theoretically predict the structure of a system of spherical fillers immersed in physically and chemically heterogeneous polymeric materials.

The aim of the present work is to theoretically explore the electrical response of the DBC-particle composites depending on filler affinities for copolymer blocks and the interactions between fillers. Technically, this aim is achieved by combining the phase-field modeling of three-dimensional morphologies of the microphase separated DBC system, Monte-Carlo simulations that determine equilibrium distribution of fillers in the predicted DBC morphologies, and resistor network model that calculates a resulting conductivity of the composite. We investigate how the conductivity of the composite depends on the difference between the affinities of fillers for dissimilar copolymer blocks at a given morphology of the DBC system. Note that the described theoretical method can be applied to a whole variety of morphologies of filled DBC systems not restricted to the specific case of the lamella morphology considered in the present work. Recall that asymmetric DBC system is known to assume, in particular, cylindrical morphology both in bulk and in thin films [25]. Similarly to the lamellae, the cylindrical microphases formed by DBC can serve as geometrically well-defined host domains for conductive fillers, thus providing conduction pathways in the DBC-particle composite. Application of the developed method to the described case of the cylindrical DBC morphology is straightforward and it will be reported elsewhere. From a broader perspective, the proposed method of the calculation of the composite conductivity can be generalized to other heterogeneous polymer-particle composites with given polymer-filler interactions.

The paper is organized as follows. In the next Section 2 we describe theoretical and simulation methods used to investigate the distribution of fillers and conductivity of the DBC-particle composite. For the convenience of a reader, this section is subdivided into several subsections that consecutively describe the physical aspects of the immersion of the fillers into the DBC system, the distribution of fillers depending on the morphology of the DBC system, and the conductivity of the composite for these distributions. In Section 3 we discuss the obtained results and show illustrative examples of the distribution and conductivity of the filler system depending on the interaction between fillers and that between fillers and polymers. In the last Section 4 we give conclusions and outlook.

## 2. Theory and Simulations

### 2.1. Energy of Immersion of a Filler into Microphase-Separated Diblock Copolymer System

Consider the incompressible symmetric DBC system containing hard spherical fillers having radius *R*. Each copolymer molecule having the polymerization degree *N*, is comprised of two blocks *A* and *B* each containing N/2 same monomers. The energy of immersion of a filler into inhomogeneous polymer system essentially depends on the relation between the entropic interactions [26] between fillers and polymers and the affinity of this filler for polymers [27]. The interplay between the above two effects determines, in particular, stability of fillers against coagulation [28] underlying their ability to form clusters (agglomerates). In the present work we use celebrated Ohta-Kawasaki model [29] to describe the effect of fillers on the thermodynamic state of the DBC system. The main advantage of this model is in its relative simplicity achieved without essential loss of the predictive power.

In the frameworks of this model, the thermodynamic state of the described incompressible symmetric DBC system is described by the free energy

(1) $$\beta F=-D\left(\int \left[\eta \nabla^{2}\eta \left(\vec{r}\right)+\xi^{-2}\eta {\left(\vec{r}\right)}^{2}\left(1-\frac{\eta {\left(\vec{r}\right)}^{2}}{2}\right)\right]dV-\lambda \xi^{-4}\int dV\int \frac{\eta \left(\vec{r}\right)\eta \left({\vec{r}}_{1}\right)}{4\pi |\vec{r}-\vec{r_{1}}|}dV_{1}\right).$$

Here, $\vec{r}$ (${\vec{r}}_{1}$) is the position vector and dV ($dV_{1}$) is the corresponding volume element, $\beta ={\left(kT\right)}^{-1}$ is the reciprocal temperature, *k* and *T* being the Boltzmann constant and the absolute temperature, respectively; $D=R_{G}^{2}/\left(4v_{0}N\right)$, $\eta =2v_{0}\rho_{A}-1\left(=1-2v_{0}\rho_{B}\right)$ is the reduced density of polymer species *A* (*B*), $v_{0}$ being the specific monomer volume that is assumed to be equal for species *A* and *B*, χ and α=χN are the Flory-Huggins and segregation parameters, respectively, $\xi^{2}\equiv =R_{G}^{2}/\left(\alpha -3.6\right)$ is the correlation length that quantifies a typical width of local compositional non-uniformities in the system (e.g., interfaces between *A*-rich and *B*-rich polymer phases), $R_{G}$ is the gyration radius of copolymers. Note that the order parameter η varies between the limits 1 and −1 corresponding to the pure *A* and *B* polymer phases, respectively. The last term in the right hand side (r.h.s.) of Equation (1) describes the long-range compositional correlations that cause microphase separation of the DBC system into *A*-rich and *B*-rich polymer lamella phases that occurs at α∼10.5. The coefficient λ of this term equals to $12\xi^{4}/R_{G}^{4}$.

The equilibrium morphology of the copolymer system is determined by the minimization of the grand canonical potential. For the pure symmetric DBC system, Ω equals to the Helmholz free energy defined by Equation (1). This is because the chemical potentials $\mu_{A}$, $\mu_{B}$ of the polymer species *A* and *B* are equal, so that their difference does not contribute to the Gibbs part $G={\left(2v_{0}\right)}^{-1}\int \left(\mu_{A}-\mu_{B}\right)\eta dV$ of the grand potential of *incompressible* copolymer system. Note that in the presence of external fields $w_{A}$, $w_{B}$ acting on *A*- and *B*-blocks, the above Gibbs correction to the free energy is non-zero and it equals to

(2) $${\left(2v_{0}\right)}^{-1}\int \left(w_{A}-w_{B}\right)\eta dV.$$

In what follows we will use the external fields $w_{A,B}$ and the associated Gibbs correction to describe the effect of the polymer-particle interaction on the composition of the DBC in the vicinity of these particles.

The minimization of the free energy given by Equation (1) with respect to η leads to the following equation for the order parameter $\eta_{b}$ of the pure diblock copolymer system

(3) $$-\nabla^{2}\eta_{b}-\xi^{-2}\eta_{b}\left(1-\eta_{b}^{2}\right)+\xi^{-4}\lambda J\left[\eta_{b}\right]=0,\;J\left[\eta \left(\vec{r}\right)\right]=\int \frac{\eta ({\vec{r}}_{1})}{4\pi |\vec{r}-\vec{r_{1}}|}dV_{1}.$$

The obtained Equation (3) can be further simplified by applying the Laplacian to its l.h.s., which leads to equation

(4) $$\nabla^{2}\left(\nabla^{2}\eta_{b}+\xi^{-2}\eta_{b}\left(1-\eta_{b}^{2}\right)\right)+\lambda \xi^{-4}\eta_{b}=0.$$

**Figure 1:**
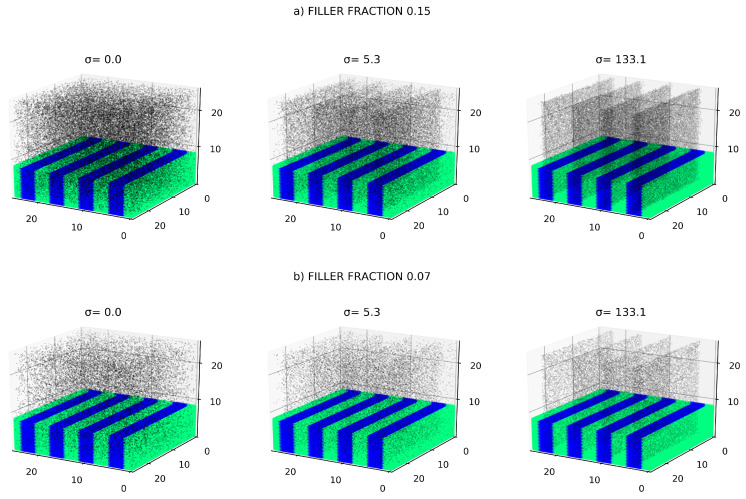
Effect of the contrast between the affinities of fillers for dissimilar copolymer blocks on the dictribution of these fillers in the microphase-separated DBC system: (**a**) larger filler volume fraction ϕ=0.15; (**b**) smaller filler volume fraction ϕ=0.07. Black dots show the centers of the filler particles. The upper part of the image of the host DBC matrix is cut off to make visible the distribution of fillers inside the DBC system. Selective *A* phase having larger affinity for polymers is shown in blue, *B* phase in green. All lengths are measured in $R_{G}$.

**Figure 2:**
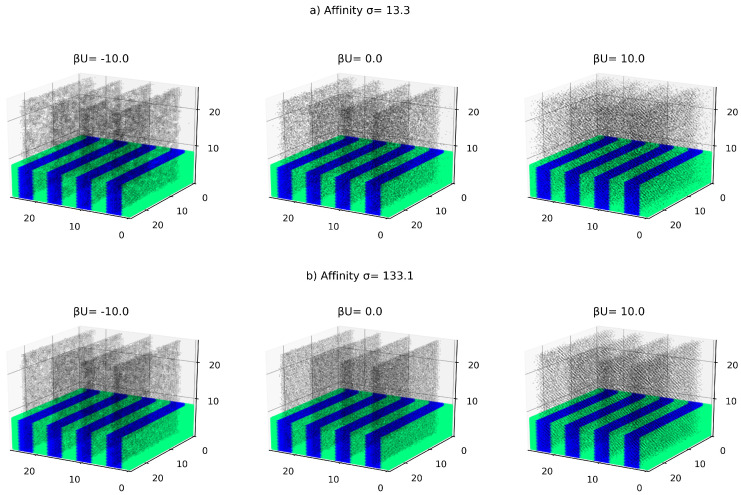
Effect of the interaction between fillers on the dictribution of fillers in the microphase separated DBC system: (**a**) smaller contrast between the affinities of fillers for dissimilar copolymer blocks σ=13.3; (**b**) larger contrast between the affinities of fillers for dissimilar copolymer blocks σ=133.1. The volume fraction of particles is ϕ=0.15. Black dots show the centers of the filler particles. Selective *A* phase having larger affinity for polymers is shown in blue, *B* phase in green. The upper part of the image of the host DBC matrix is cut off to make visible the distribution of fillers inside the DBC system. All lengths are measured in $R_{G}$.

The obtained Equation (4) is known [30,31] to properly describe the transition from the random state of the DBC system to the ordered lammelae morphology. Mathematically, this transition occurs when control parameter λ reaches the critical value of 0.25. Periodic solutions of Equation (4) describing the lammela morphology of the DBC system exist for the values of λ lying between 0 and 0.25. Upon exceeding the value of 0.25, the copolymer system switches into the random state described by the uniform solution $\eta_{b}=0$. An example of the lamella morphology obtained from Equation (4) is given in Figure 1 and Figure 2 that show the solution of Equation (4) for λ=0.18. This solution describing the morphology of the DBC system in the considered case has been obtained by applying the iso-geometric finite difference numerical method implemented in the PetIGA project [32].

The presence of nano-particle fillers changes the thermodynamic state of the described pure DBC system. In order to describe these changes consider the immersion energy of a single filler of radius *R* into this system. The entropic and enthalpic interactions between the particle surface and copolymers cause the formation of the polymer inter-phase in the vicinity of this particle. The compositional structure of this interface is described by equations similar to (3) with the only difference that the quantity $\mu_{A}-\mu_{B}$ is no longer zero in the presence of fields $w_{A,B}$ describing the polymer-particle interactions. In the presence of particles, the equation for η therefore becomes

(5) $$\partial_{r^{2}}^{2}\eta \left(r\right)+2r^{-1}\partial_{r}\eta \left(r\right)+\xi^{-2}\eta \left(r\right)\left(1-\eta {\left(r\right)}^{2}\right)-\lambda \xi^{-4}J\left[\eta \left(r\right)\right]=w\left(\vec{r}\right),$$

where the origin of the coordinate frame is the particle center so that $r\equiv |\vec{r}|$ measures the distance from the filler particle center to the point with position vector $\vec{r}$. The quantity $w\left(\vec{r}\right)\equiv {\left(4Dv_{0}\right)}^{-1}\beta \left(w_{A}\left(\vec{r}\right)-w_{B}\left(\vec{r}\right)\right)$ in the r.h.s. of Equation (5) is proportional to the total external field acting on the order parameter of the copolymer system. This field describes the overall effect of the particle on the incompressible DBC system. Note that for the considered incompressible DBC system, it is the difference of the interactions between the particle and dissimilar polymer blocks that changes the local composition of the DBC system in the vicinity of the particle relative to that in the bulk.

Substituting the expression for *w* given by the l.h.s. of Equation (3) into the grand canonical potential Ω=F−G given by Equations (1) and (2) one obtains the expression for the equilibrium grand potential $\Omega_{eq}$ of the DBC system in the presence of particles. Subtracting the counterpart of the obtained expression for Ω for pure diblock copolymers, one obtains the expression for the excess grand potential caused by the presence of a particle

(6) $$\beta \Delta \Omega_{eq}=D\int \eta \left(\vec{r}\right)w\left(\vec{r}\right)dV-\frac{D}{2\xi^{2}}\int \left[\eta {\left(\vec{r}\right)}^{4}-\eta_{b}{\left(\vec{r}\right)}^{4}\right]dV.$$

Note that the obtained expression for the excess potential Ω contains two terms. The first term in the r.h.s. of Equation (6) that is hereafter denoted by $\beta F_{S}$, describes the direct adsorption (enthalpic) interactions between the particle and copolymers. The second term describes the osmotic effect caused by the particle on the DBC system. In what follows, we separately evaluate these contributions for the considered case of strongly segregated incompressible diblock copolymer system.

The first enthalpic term in the r.h.s. of Equation (6) describing the interaction between the particle surfaces and the copolymers can be evaluated by adopting realistic approximation that the range of the considered weak adsorption interactions is of the order of the monomer length. Note that this assumption rules out the presence of the long-ranged electrostatic interactions between the particles and polymers that are not covered by the present theory. Mathematically, the described approximation can be expressed using the delta-functional form of the potentials $w_{i}=-w_{i}^{0}b\delta (|\vec{r}-{\vec{r}}_{O}\left|-R\right)$, where ${\vec{r}}_{O}$ is the position vector of the particle center. *b* and $w_{i}^{0}$ describe the range and strengths of the adsorption potentials, respectively. The described approximation brings the above expression for the adsorption term into the form

(7) $$F_{S}\equiv {\left(4v_{0}\right)}^{-1}\int \eta \left(r\right)\left(w_{A}\left(\vec{r}\right)-w_{B}\left(\vec{r}\right)\right)dV=-2^{-1}R^{2}\left(\gamma_{A}-\gamma_{B}\right)\int_{\Omega }\eta \left({\vec{r}}_{O}+R\vec{n}\right)d\vec{n},$$

where $\vec{n}$ is the unit vector directed from the center of the spherical particle to the point of particle surface described by the body angle Ω, and the integration is over the full body angle. $\gamma_{i}\equiv {\left(2v_{0}\right)}^{-1}w_{i}^{0}b$ is the surface energy (adhesion energy per unit area) of the copolymer block i=A,B. Due to the described approximation of the short-ranged adsorption potential, the expression for the surface part of the particle immersion energy factorizes into the two multipliers. The first multiplier is the integral that depends only on the position of the particle surface in a non-uniform DBC system. The second multiplier is the position-independent coefficient that quantifies the difference between the affinities of the particle for copolymer blocks *A* and *B*. The latter term can be evaluated from the adhesion energies of the polymer-filler pairs measured in experiments [33] that prove that $\gamma_{i}$ are different for different filler-polymer pairs, being of the order of dozens of $mJ/m^{2}$. In the present work we use the reduced surface energy $\sigma =4\pi R^{2}\beta \left(\gamma_{A}-\gamma_{B}\right)$ as a dimensionless parameter quantifying the total effect of the enthalpic interactions between the particle surface and the DBC system. As this parameter is inversely proportional to the temperature, it can be varied in a wide range by changing the thermodynamic conditions.

The second osmotic term in the r.h.s. of Equation (6) depends on the structure of the above described polymer-particle inter-phase. In the considered case of strongly segregated incompressible DBC system, the morphology of this system consists of the alternating lamellae of *A* and *B* blocks having almost uniform density and composition. These domains are separated by the interfaces having a width of the order of the correlation length ξ [29]. ξ in turn evaluates to ∼5 nm, i.e., it is of the order or less than the nanoparticle radius. Under the described conditions, it is therefore reasonable to neglect the effect of particles placed within the domains of almost pure *A* and *B* phases on the compositional structure of the DBC system in the vicinity of these particles. These physical conditions are described by the exact trivial solutions η=±1 of Equation (5) corresponding to pure *A* (η=1) and *B* (η=−1) polymer phases. It is important to note that even this simple solution does not imply the vanishing of the second term in the r.h.s. of Equation (6). This is because of the excluded volume effect caused by the fact that the copolymers are expelled from the volume occupied by the particle. Mathematically, this effect is expressed by the fact that the quantity $\eta^{4}-\eta_{b}^{4}$ that enters the second term in the r.h.s. of Equation (6) vanishes everywhere apart from the particle interior, where it equals to $-\eta_{b}^{4}$. Adding the described osmotic part of the immersion energy to the surface part given by Equation (7) results in the final expression for the particle immersion energy given by

(8) $$\beta \Delta \Omega =-\frac{\sigma }{8\pi }\int_{\Omega }\eta_{b}\left({\vec{r}}_{O}+R\vec{n}\right)d\vec{n}+\frac{D\xi^{-2}}{2}\int_{0}^{R}dss^{2}\int_{\Omega }\eta_{b}{\left({\vec{r}}_{O}+s\vec{n}\right)}^{4}d\vec{n}.$$

The obtained quantity ΔΩ is of key importance for determining the distribution of particles in the DBC system discussed in the next section.

### 2.2. Distribution of Fillers in the DBC System

The distribution of fillers in the micro-phase-separated DBC system is primarily determined by the two main factors. The first factor stems from the enthalpic (adsorption) and osmotic interactions between the filler particles and polymers and the second factor arises from the interaction between these particles. In the case when the particles have different affinities for dissimilar copolymer blocks, the first factor is position-dependent. This observation is explained by the following arguments. Recall that the minimal work quantifying the overall enthalpic and entropic cost of immersion of a particle in the equilibrium DBC system at given thermodynamic conditions is fully determined by the immersion energy ΔΩ calculated in the preceding section. According to the Widom theorem [34], the probability that a particle is placed in the position where it produces the excess energy ΔΩ is proportional to exp(−βΔΩ). The quantity ΔΩ, in turn, depends on the local morphology of the DBC system. As will be shown in what follows, in the considered case of dilute to moderate concentrations of fillers (up to 20% volume fraction) in the incompressible DBC system, the described factor plays a dominant role in the distribution of fillers in this system.

Recall that the described enthalpic factor consists of two parts expressed by the corresponding terms in the r.h.s. of Equation (8). The first term describes the direct adsorption interactions between the particles and copolymer blocks. This term is odd in the order parameter η and therefore favors the placement of the particles in the phase *A* that has larger affinity for these particles. In contrast, the second, osmotic term that is even in η, depends only on the local compositional contrast (i.e., the absolute value of the deviation of the local fractions of *A*-blocks from their mean). This term favors the placement of the particles in the interfaces between lamella domains, where |η| is minimal. Physically, this term describes the tendency that the fillers located at the interfaces screen energetically unfavorable contacts between dissimilar blocks *A* and *B*.

The second above mentioned important factor that affects the distribution of fillers in the DBC system is the interaction between fillers. This interaction consists of the three components as follows. The first component is the short-ranged steric (excluded volume) interaction between the filler particles. The second component is the direct molecular (e.g., van-der-Waals) interaction between the particle surfaces. The third component is the polymer-mediated interactions between fillers [35,36]. For the considered case of the incompressible DBC system, the main osmotic component of these interactions is suppressed, as it relies [37] on finite compressibility of a polymer system. As has been recently shown [35,36], there is an additional weak long-ranged component of the polymer-mediated interactions present in binary polymer systems that relies on the compositional fluctuations in these systems. This component is also suppressed when particles are placed within the lamella domains of the strongly-segregated DBC system that have almost uniform composition. The described compositional mechanism of the polymer-mediated interactions therefore takes place only in the interfacial regions of the DBC system that are non-uniform in polymer composition. In the case of strong segregation of the copolymer blocks considered in the present work, this effect can be also neglected, as a typical width ξ of these interfaces is of the order of several nanometers [38], i.e., of the order or less than the realistic size of nano-particles used in practice (10–1000 nm).

A realistic minimal model of the inter-particle interactions in the considered case of incompressible strongly-segregated DBC system must therefore include only the described position-independent steric and van-der-Waals interactions. A more refined model taking into account the polymer-mediated interactions might be necessary to properly describe the case of a weakly-segregated DBC system, where the described effect of the compositional fluctuations is important. This model will be reported elsewhere.

The described effects of the local composition of the DBC system and the interactions between particles on their distribution are captured by the proposed Monte-Carlo lattice model described in what follows. The considered system is the microphase-separated filled DBC depicted in Figure 1 and Figure 2, which is comprised of several (9 in the picture) lamella domains. Recall that these domains are formed when the segregation parameter α is sufficiently large and 0<λ<0.25, as can be deduced from the solutions of Equation (3). The morphology of the DBC system is represented by continuous field of the order parameter η obtained from the finite element analysis described in Section 2.1. The smallest length scale in the system is determined by the radius *R* of the nano-particles that is set equal to 0.19$R_{G}$. Note that in the considered segregated DBC system that consists of well separated lamella domains, it proves to be convenient to measure the radius of the nano-particles in terms of the domain length *L*, which gives R=0.03L. The system is subdivided into the cubic cells with side lengths 2R that can potentially contain only one nano-particle.

The equilibrium distribution of fillers in the described cubic lattice that represents the described DBC lamella system is determined by the Metropolis Monte-Carlo simulations [39]. The interaction energy that determines the probability of placement of a particle into a given cell consists of two contributions. The first contribution stems from a standard position-independent pair interaction potential *U* acting between the neighboring particles. This contribution, outlined in the preceding paragraph, describes the molecular (e.g., van-der-Waals) interactions between fillers. Note that we consider both cases of attractive and repulsive interactions having strength varied in range −10kT<U<10kT, as described in what follows.

The second contribution is caused by the interaction between the particles and DBC system. This contribution is determined by the immersion energy ΔΩ given by Equation (8). As is explained in the preceding section in detail, ΔΩ contains the surface and volume terms represented by the respective surface and volume integrals in the r.h.s. of Equation (8). Note that in the considered case where the composition of the DBC system having the lamella morphology depends on the only coordinate *x* perpendicular to the lamella domains, the calculation of the above integrals greatly simplifies. This calculation is performed numerically for each possible particle position, by using the order parameter field $\eta (\vec{r})$ obtained by the finite element analysis of Equation (3).

The calculated distribution of conductive particles depending on the surface energy determines the conductivity of the composite calculated in the next section.

### 2.3. Conductivity of the Filled DBC System Depending on the Distribution of Fillers

In order to calculate the conductivity of the DBC-particle composite we use the lattice model described in the preceding section. The distribution of particles in cells of the lattice representing the non-uniform DBC system obtained from each run of the Monte Carlo simulations described in Section 2.2 provides an input of the composite conductivity calculation. The problem is therefore reduced to calculating the conductivity of the system of particles that occupy cells of the regular cubic lattice.

To evaluate the conductivity of the composite modeled by the above lattice system, one needs to evaluate the resistance (conductance) of a pair of filler particles that are in contact. In realistic polymer-particle composites, this conductance can be caused [13,14] by unconventional physical mechanisms, such as constriction and hopping conductivity, not restricted to the standard contact conductivity. Because of physical diversity of possible conductivity mechanisms that can vary for different polymer-particle systems, as well as low experimental accessibility of the conductivity of nanoscopic particles, we chose different route for practical evaluation of the composite conductivity. Specifically, instead of relying on the elementary conductance of a pair of fillers in contact as known, we relate the simulated conductance to the conductance of the completely filled lattice $s_{0}$ (as described by the filler volume fraction ϕ=1). The thus obtained quantity $S=s/s_{0}$ evaluates the reduced conductivity corresponding to a given volume fraction of fillers, provided that the completely filled counterpart of the described partially filled lattice has the same size. The conductivity ∼$s_{0}$ of the completely filled lattice can be approximately identified with the conductivity of the bulk filler material well known for majority of commonly used fillers.

To calculate the conductivity, we set thought parallel electrodes separated by a distance of 25 $R_{G}$ that impose a constant voltage on the composite. For the sake of definiteness, we restrict ourselves to the consideration of more common perpendicular orientation of the described DBC lamella domains with respect to the surfaces of these electrodes. Recall that the considered perpendicular lamella orientation proves [40,41,42,43] to be entropically favorable for a free DBC system in the absence of the enthalpic interactions between the DBCs and confining walls. This fact ensures practical applicability of the selected morphology of the simulated system. Note that the presence of the above enthalpic interactions between the confining walls and polymers is known to enforce [40,41] the parallel lamella orientation. Even when formed under these energetically favorable conditions, the described parallel orientation flips [41,43] to the perpendicular one when the natural period of the lamellae is incommensurate with the separation between the confining walls. Generally, the desirable orientation of the DBC lamella domains can be always achieved [44] by the directed self-assembly at the patterned substrates, which in turn can be used to affect the distribution of particles and resulting conductivity of the composite.

The conductance of the described cubic lattice comprised of cells irregularly filled with conductive particles is calculated by combining the resistor network model and the site percolation lattice model described in what follows. The sites of the conducting lattice are identified by all possible positions of the centers of the filler particles. When two neighboring sites of the conductive lattice are occupied, elementary conductance *c* is assigned to the bond connecting these sites. The zero conductance is assigned to the rest of the lattice bonds not connecting two occupied lattice sites. It is convenient to consider the described conductive cubic lattice as a stack of the square lattices formed by the “horizontal” layers of this lattice parallel to the electrodes. The layers are enumerated by incrementing index *l*, so that the negative and positive electrodes correspond to 0th and *L*th layers, respectively. The effect of the presence of the conductive bond attached to a given site (i,j) of the layer *l* causes different effect on the overall conductivity of the lattice depending on the orientation of this bond with respect to the electrodes. It is therefore convenient to distinguish between the “vertical”-*z* bonds oriented perpendicularly to the electrodes and having conductance $v_{ij}$, and the “horizontal-*x*” (“horizontal-*y*”) in-plane bonds lying in the layer *l* and having conductance $h_{ij}$ ($g_{ij}$). Recall that the above conductances $v_{ij}$, $h_{ij}$ and $g_{ij}$ can assume only two values *c* and 0 for conductive and insulating bonds, respectively.

Immediate application of the Kirchhoff’s laws to (i,j)th site of the layer *l* of the conductive lattice gives the following iteration relations among currents $I_{ij}^{l}$ and voltages $U_{ij}^{l}$ at site (i,j) and those at the neighboring sites

(9) $$\begin{array}{c} U_{ij}^{l}=U_{ij}^{l+1}-v_{ij}^{l-1}I_{ij}^{l+1},\;\\ I_{jk}^{l+1}=I_{jk}^{l}+\sum_{i}V_{jk}^{il}\left[h\right]U_{ik}^{l}+\sum_{i}V_{kj}^{il}\left[g\right]U_{ji}^{l}, \end{array}$$

where $\delta_{i}^{j}$ is the Kronecker’s delta symbol and $V_{jk}^{il}\left[h\right]=\delta_{i}^{j}\left(h_{(j-1)k}^{l}+h_{jk}^{l}\right)-\delta_{i}^{j-1}h_{(j-1)k}^{l}-\delta_{i}^{j+1}h_{jk}^{l}$.

A set of the obtained linear equations is solved numerically using the “boundary” conditions $U_{ij}^{0}=0$ at the negative electrode and $U_{ij}^{L}=U$ at the positive electrode. The resulting current *I* through the system and its total conductance *s* are obtained as $I=\sum_{i,j}I_{jk}^{L}$ and s=I/U, respectively.

## 3. Results and Discussion

### 3.1. Distribution of Fillers in DBC System

The results of the calculation described in Section 2.2 is illustrated in Figure 1 and Figure 2 that show the distribution of fillers in the DBC system for selected affinities of the fillers for polymers and strength of the interaction between these fillers.

Figure 1 illustrates the effect of the contrast between the affinities of fillers for dissimilar copolymer blocks on the distribution of these fillers in the microphase separated DBC system. The interaction between fillers is set equal to kT for all the investigated cases shown in Figure 1. This interaction models slight repulsion between closely spaced fillers that can be caused by the repulsive steric or close-distance van-der-Waals interactions. These repulsive interactions slightly suppress the crowding of the fillers. Note that according to our simulation results, the interaction between fillers *U* can significantly affect their distribution only when |U|⪆kT. The cases illustrated by Figure 1 therefore correspond to the weak interaction between fillers not having essential effect on their distribution. The effect of significant attractive and repulsive inter-particle interactions on the distribution of fillers is considered in the second part of the current subsection (see Figure 2 with the explanations to this Figure below).

The simulated system depicted in Figure 1 and Figure 2 consist of several alternating lamella layers comprised of almost pure polymer phases *A* and *B* filled with conductive particles. The average concentration of fillers is controlled by their overall volume fraction ϕ that assumes the values 0.05, 0.07, 0.1, 0.15, and 0.20. The reduced segregation parameter λ of DBC defined below Equation (1) is set equal to 0.18 that corresponds to the intermediate segregation regime closer to the strong segregation extreme. The contrast $\Delta \gamma \equiv \gamma_{A}-\gamma_{B}$ between the affinities of the fillers for dissimilar copolymer blocks is set constant for each specific run of the simulations. Recall that this affinity contrast is quantified by the surface energy σ∼Δγ defined below Equation (7). σ is incrementally increased from 0 to 300 and the corresponding distribution of fillers is determined by the Monte Carlo procedure as described in Section 2.2. The illustrative examples of the distribution of filler particles is shown for two different volume fractions 0.15 (upper panel *a*) and 0.07 (lower panel *b*). As can be elucidated from Figure 1, increasing σ causes more pronounced localization of the fillers in the selective *A*-domains that have larger affinity for these fillers.

The observed localization effect stems from the fact that σ is proportional to the difference between the affinities $\gamma_{A}$ and $\gamma_{B}$ of the fillers for polymer species *A* and *B*. Therefore, this parameter favors the placement of the particles into the selective *A*-domains. Note that the surface interactions described by the parameter σ play a dominant role in the effect of the DBC morphology on the filler distribution. This is because the additional osmotic contribution described by the second volume integral in the r.h.s. of Equation (6) is of the order of $\eta^{4}$. In the considered case λ=0.18, |η| is less than 0.45 in the whole spatial region. The described surface energy term ∼σ that is of the order of η therefore dominates over the above osmotic term $∼\eta^{4}$. Still, the latter osmotic (volume) term can give considerable contribution at smaller contrast between affinities of the fillers for dissimilar copolymer blocks in the case σ≪1, and cannot be neglected.

Note that for weak interactions between particles, the concentration of particles has rather minor effect on their relative distribution. This fact can be elucidated by comparing the upper and lower panels of Figure 1 that show three different cases of the distribution of particles having the same affinities for copolymer blocks in each case. The upper panel *a* shows the filler distributions for larger volume fraction 0.15, while the lower panel shows the distributions for smaller volume fraction 0.07. It can be seen that the relative fraction of particles located in the selective domains of the DBC system only slightly depends on their overall volume fraction ϕ, being mainly determined by the affinity contrast of the particles for copolymer blocks.

The above described simulation results represented by Figure 1 therefore lead to qualitative conclusion that the affinity contrast ∼σ is a decisive factor determining the distribution of fillers in the DBC system when the inter-particle interactions are relatively weak (U⪅kT). Increasing σ is shown to result in that the particles become mainly localized in the selective *A*-domains, the more, the larger the affinity contrast ∼σ. The presence of this localization effect only slightly depends on the overall particle volume fraction.

An additional effect on the distribution of filler particles in the DBC system relies on the interaction between these particles. When this interaction is large enough |U|⪆kT, it can play an essential role in the distribution of fillers. This effect is illustrated in Figure 2. This Figure shows the distribution of particles for different strengths and sign of the inter-particle interaction ranging from strongly repulsive interaction (U∼10kT) to strongly attractive one (U∼−10kT). As can be elucidated from this Figure, the attractive interactions between the fillers plays in favor of more pronounced localization of these fillers in the selective *A*-domains. It is important to note that the considered effect of the short-ranged inter-particle interactions is secondary to the enthalpic effect of the filler-polymer interactions described in the preceding paragraph. This is because the considered effect of the inter-particle interactions can manifest itself only at sufficiently large local concentration of particles when the probability of contact among these particles is sufficiently high. As is explained in the preceding paragraphs, the increase in the local concentration of fillers occurs as a result of larger affinity of these fillers for one of the copolymer blocks. In the domains of the increased local concentration of fillers the contribution of the short-ranged inter-particle interactions to the system energy is larger, as the fillers in these domains are separated by smaller distances. The inter-particle attraction therefore enhances the described effect of gathering the particles in the selective domains of the phase-separated DBC system, which lowers the system enthalpy. The same mechanism suppresses crowding the particles in the selective domains when the inter-particle interaction is repulsive.

### 3.2. Conductivity of the Filled DBC System

The distribution of fillers affected by their affinities for copolymer blocks investigated in the preceding section determines the conductance of the composite. According to the results of Section 3.1, when the contrast between the affinities of the fillers for dissimilar copolymer blocks is small, the distribution of these fillers in the DBC system is almost homogeneous. In this case, the only factor that affects the filler distribution is the tendency to screen energetically unfavorable contacts between the dissimilar copolymer blocks at the interfaces. In the considered case of strongly segregated DBC system this effect is found to be not pronounced enough to cause an essentially non-uniform distribution of fillers. For relatively low volume fractions of fillers considered in the present work (up to ca. 20%), the observed at σ<<1 almost homogeneous distribution of fillers cannot provide finite conductivity of the composite. This is because the number of contacts among these fillers is insufficient to cause the percolation of current through these contacts.

**Figure 3:**
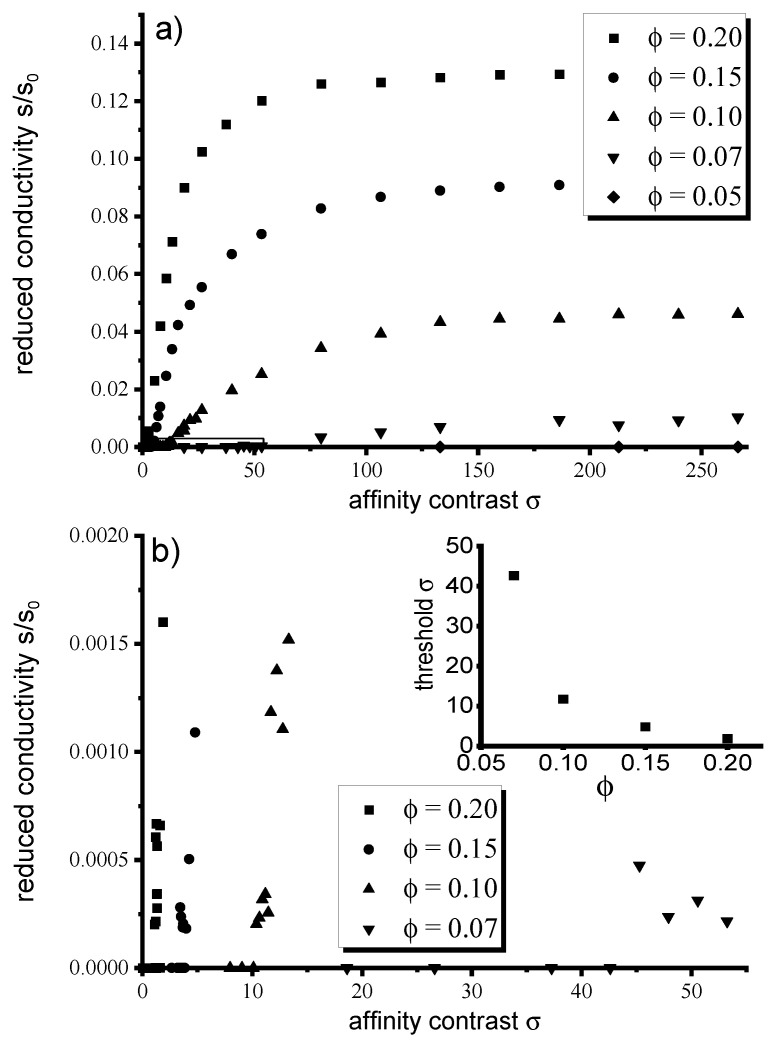
(**a**) Effect of the contrast between affinities of the fillers for dissimilar copolymer blocks quantified by σ on the conductivity of the DBC composite for several selected volume fractions ϕ of the fillers. The interaction between fillers is set equal to kT. (**b**) Zoom of the highlighted portion of Figure (**a**) in the upper panel. The inset shows the threshold values of σ delineating the conductive and insulating states of the composite.

With increasing the affinity contrast Δγ∼σ of the fillers for dissimilar polymer blocks, these fillers tend to be localized in the selective domains of the DBC system. This effect is described in the preceding subsection in detail. The described localization of fillers promotes increasing the local density of these fillers in the selective domains, as well as the number of contacts among them. Upon reaching a certain threshold value of σ, specific to each filler volume fraction, the number of contacts among fillers becomes sufficient to overcome the current percolation threshold. In the case of the considered lamella morphology, this effect results in the occurrence of current in response to the electrical potential applied in the direction parallel to the lamellae. The described transition between the insulating and conducting states of the composite upon increasing the affinity contrast Δγ∼σ can be quantitatively elucidated from Figure 3. This Figure shows the conductivity of the DBC composite as a function of the affinity contrast σ for several values of the particle volume fraction ϕ.

According to the results of Section 3.1, the degree of the localization of fillers within the selective domains of the DBC system is controlled by the affinity contrast Δγ∼σ that changes in range 0<σ<275 in Figure 3. The overall volume fraction ϕ is set equal to 0.05, 0.07, 0.1, 0.15, and 0.20 in the consecutive simulation runs. The corresponding cases are depicted by different curves on Figure 3. Recall that Figure 3 describes the case of the weak inter-particle interaction that is set equal to kT, which corresponds to the distribution of fillers depicted in Figure 1 in the preceding section.

Figure 3 shows that the conductivity of the composite containing 0.05 fraction of fillers is negligible in the whole investigated range of σ. The rest of the curves describing larger filler volume fractions 0.07, 0.1, 0.15, 0.20 show similar qualitative trends, differing only in quantitative details. Increasing the affinity contrast Δγ from zero to some threshold value specific to each filler volume fraction ϕ has no effect on the conductivity so that the composite remains insulating. Further increase of Δγ above this threshold value first results in a rapid increase of the conductivity at the intermediate values of σ∼Δγ and then the saturation of the conductivity to a limiting value specific to each filler volume fraction ϕ. The lower panel *b* of Figure 3 shows the zoomed portion of its upper panel *a*, to make visible the behavior of the reduced conductivity close to the insulator-conductor transition. The inset of this panel shows the threshold values of σ delineating the transition from the insulating to conducting states of the composite. As can be elucidated from this inset, the threshold value of σ steeply decreases with increasing the filler volume fraction ϕ. This observation corroborates the above qualitative conclusion that the conductor-insulator transition at smaller overall filler volume fractions occurs at larger affinity contrast Δγ of these fillers for dissimilar copolymer blocks.

**Figure 4:**
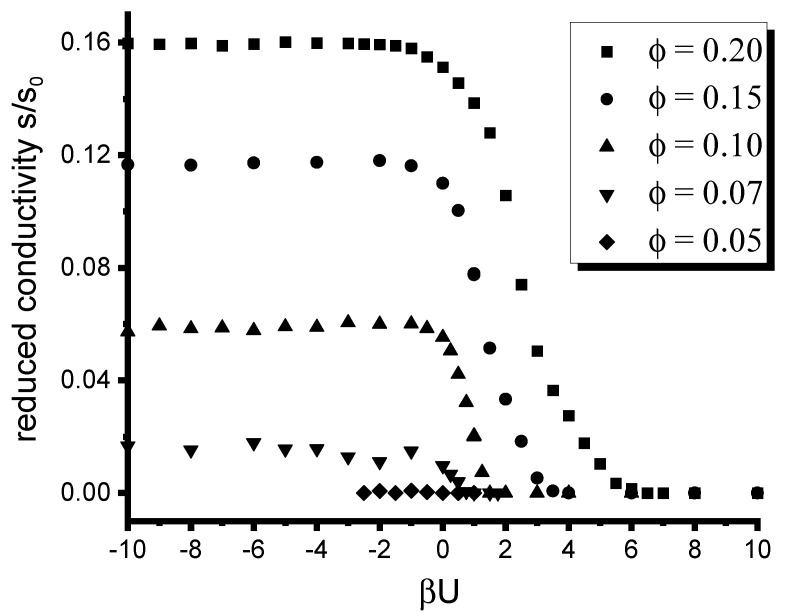
Effect of the interaction between fillers on the conductivity of the filled microphase-separated DBC system for several selected volume fractions of fillers. σ is set equal to 133.1.

**Figure 5:**
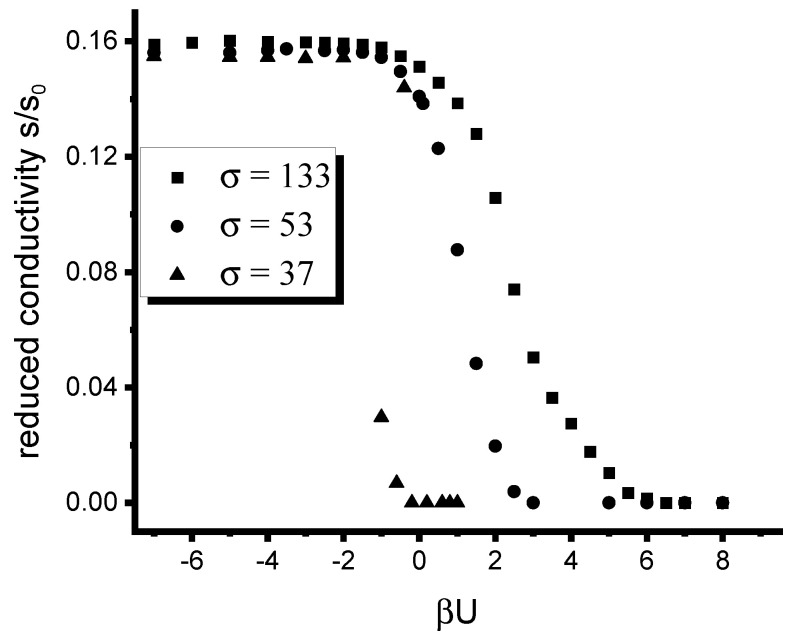
Effect of the interaction between fillers on the conductivity of the filled microphase-separated DBC system for several selected values of the affinity contrast σ∼Δγ. The volume fraction of particles ϕ is set equal to 0.2.

Figure 4 and Figure 5 illustrate the effect of the inter-particle interactions on the conductivity of the composite. Figure 4 shows the dependence of the reduced conductivity of the composite on the reduced inter-particle potential βU for the same set of values of the filler volume fraction as that used in Figure 3. The quantity σ∼Δγ that describes the contrast between affinities of the fillers for dissimilar copolymer blocks is set equal to 133.1. As can be elucidated from Figure 4, the inter-particle interaction plays an important role in the distribution of fillers only for larger volume fractions ϕ>0.1. For smaller ϕ≤0.1, the inter-particle interactions cause a considerable effect on the conductivity only in the region U⪅−kT. Note that this observation is in full agreement with Figure 2 illustrating the distribution of particles depending on βU. Specifically, the inter-particle interaction significantly affects the conductivity of the composite only in the region of values of βU where one observes stronger localization of fillers within the selective DBC domains. Note that the conductivity observed for ϕ=0.05 is negligibly small even at strong inter-particle attraction. This observation is explained by the fact that even strong localization of insufficiently small amount of fillers does not suffice to provide for the current percolation in the composite. The conductor-insulator transition for this case is observed at unrealistically high threshold values of σ=1065.

Figure 5 illustrates the effect of the inter-particle interactions on the conductivity of the composite for selected values of the affinity contrast Δγ∼σ. The filler volume fraction is set equal to 0.2. As can be elucidated form this Figure, the inter-particle interactions |U|>kT have pronounced effect on the conductivity of the composite containing relatively large volume fraction of fillers at all investigated values of σ quantifying the affinity contrast. The presence of relatively strong repulsive interactions U⪆6kT drastically suppresses the conductivity of the composite. This fact is in agreement with the observations described below Figure 2 that sufficiently strong repulsive interactions between the particles prevent their crowding in the selective domains of the DBC system. In the opposite case of sufficiently strong attractive interactions U⪅−2kT between the particles, the conductivity saturates to its limiting value specific to a given volume fraction. Again, as is explained below Figure 2, the attractive interactions between the particles enhance their crowding in the selective DBC domains, which promotes the conductivity. An additional interesting effect that can be observed from Figure 5 is that the conductivity of the filler system having smaller affinity contrast for the copolymer blocks is very sensitive to the inter-particle interactions. This fact can be observed for the case σ=37 shown in Figure 5 by elucidating the steep drop of the conductivity upon crossing from negative to positive values of βU.

## 4. Conclusions

In the present work we have investigated the conductivity of the insulating diblock copolymer (DBC) system filled with spherical conductive fillers depending on the affinities of these fillers for copolymer blocks. The distribution of fillers is found to be determined by the interaction between fillers and the contrast between the affinities of these fillers for dissimilar copolymer blocks quantified by the parameter σ. σ, in turn, is determined by the surface part of the immersion energy of a filler given by Equation (7). This immersion energy contains the additional osmotic term that results from the excluded volume (steric) interactions between filler particles and polymers. This osmotic term occurs to be negligibly small for the considered case of the incompressible strongly segregated DBC system having spatially independent polymer osmotic pressure.

Further, we have determined the equilibrium distribution of fillers by performing Monte-Carlo simulations relying on the above inter-particle interactions between fillers and the local immersion energy of these fillers. These simulations show that the fillers tend to be localized within selective domains of the phase-separated DBC system that have larger affinity for these fillers. Typical results of these simulations for different volume fractions of fillers, their affinity contrast for dissimilar copolymer blocks, and the interaction between fillers are shown in Figure 1 and Figure 2. The described localization effect promotes increasing the local polymer density in the selective domains of the DBC system, which in turn increases the probability of contacts among the fillers in these domains. Above a certain threshold value of the affinity contrast Δγ∼σ specific to a given volume fraction, the filler system experiences the conductivity percolation transition. This transition results in the conductor-insulator transition of the composite. The described threshold value is found to be strongly dependent on the inter-particle interaction when the volume fraction of fillers and the affinity contrast Δγ are sufficiently large. This observation is explained by the fact that the described effect of the inter-particle interactions is secondary to the effect of the polymer-particle interactions. This is because the interaction between particles is statistically significant only when the fillers have sufficient local density and are therefore placed close to each other. As is explained above, the domains of increased local filler density occur only when the affinity contrast Δγ and average volume fraction are sufficiently large.

The main conclusion of the present work is that DBC provides convenient polymer host system with easily adjustable morphology that can be effectively used for governing the distribution of conductive filler particles. Adjusting the distribution of fillers can in turn be effectively used to drive the electrical response of the DBC-particle composite. In particular, localization of particles in the selective domains of the DBC system is shown to induce the insulator-conductor transition. This effect can be potentially used for soft electrical sensors that would indicate the distribution of fillers by the electrical response of the composite. Recall that the distribution of fillers, in turn, is directly related to the morphology and thermodynamic state of the DBC system determined by the temperature and pressure in the composite. The electrical response of the DBC-particle system can therefore be calibrated to elucidate abrupt changes in temperature and pressure associated with changing the DBC morphology and distribution of fillers.

The present work contributes towards quantitative description of the relation between the morphology of the DBC-particle composite and the electrical response of this composite. The considered control factor that determines the distribution of fillers in the DBC system is the affinity of fillers for copolymer blocks. Note that this affinity can be adjusted by applying the well known method [33] of surface modification of fillers that relies on changing the surface interaction energy of fillers with polymers. Different degrees of the surface treatment of fillers can be used to affect their distribution in a given DBC system, thus affecting the electrical response of this system. The obtained theoretical results can be therefore readily applied to calibrating the electrical response of the DBC filled with nano-particles having known surface energy, which can be used for electrical sensing applications.
